# Positive correlation between hypertensive retinopathy and albuminuria in hypertensive adults

**DOI:** 10.1186/s12886-023-02807-6

**Published:** 2023-02-13

**Authors:** Jun Li, Wenbo Zhang, Liang Zhao, Jing Zhang, Haicheng She, Ying Meng, Yadi Zhang, Xiaopeng Gu, Yan Zhang, Jianping Li, Xianhui Qin, Binyan Wang, Xiping Xu, Fanfan Hou, Genfu Tang, Rongfeng Liao, Lishun Liu, Meiqing Huang, Xinlei Bai, Yong Huo, Liu Yang

**Affiliations:** 1 Department of Ophthalmology, Peking University First Hospital, No.8 Xishiku Street, Xicheng District, Beijing, 100034 China; 2 Beijing Ophthalmology and Visual Science Key Laboratory, Beijing Tongren Eye Center, Beijing Tongren Hospital, Capital Medical University, Beijing, China; 3 Department of Ophthalmology, Peking University International Hospital, Beijing, China; 4 Department of Cardiology, Peking University First Hospital, No. 8 Xishiku Street, Xicheng District, Beijing, 100034 China; 5 National Clinical Research Study Center for Kidney Disease, State Key Laboratory for Organ Failure Research; Renal Division, Nanfang Hospital, Southern Medical University, Guangzhou, China; 6 Institute of Biomedicine, Anhui Medical University, Hefei, China; 7 Beijing Advanced Innovation Center for Food Nutrition and Human Health, College of Food Science and Nutritional Engineering, China Agricultural University, Beijing, China; 8 School of Health Administration, Anhui University, Hefei, China; 9 Department of Ophthalmology, The First Affiliated Hospital of Anhui Medical University, Hefei, China; 10 Graduate School at Shenzhen, Tsinghua University, Shenzhen, China; 11 Data Management Center, Shenzhen Evergreen Medical Institute, Shenzhen, China; 12 China Pharmaceutical University, Nanjing, China

**Keywords:** Hypertension, Hypertensive retinopathy, Urinary albumin to creatinine ratio, Albuminuria

## Abstract

**Purpose:**

We investigated the association between albuminuria and hypertensive retinopathy (HR) in hypertensive adults.

**Methods:**

This was a cross-sectional subgroup analysis of data from the China Stroke Primary Prevention Trial. We enrolled 2,964 hypertensive adults in this study. Keith-Wagener-Barker stages was used to assess HR. The urinary albumin to creatinine ratio (UACR) was calculated to evaluate albuminuria.

**Results:**

HR was found in 76.6% (*n* = 2, 271) of the participants, albuminuria was found in 11.1% (*n* = 330). The UACR levels were significantly higher in subjects with HR than in those without HR (grade 1, β = 1.42, 95% confidence intervals [CI]: -0.12, 2.95, *p* = 0.070; grade 2, β = 2.62, 95% CI: 0.56, 4.67, *p* = 0.013; grade 3, β = 5.17, 95% CI: 1.13, 9.20, *p* = 0.012). In the subgroup analyses, the association between HR and UACR was stronger in current smokers (*p* for interaction = 0.014). The correlation between HR grades 1 and 2 and UACR was stronger in subjects with higher triglyceride levels (≥ 1.7 mmol/L), but for grade 3 HR, this correlation was stronger in subjects with lower triglycerides levels (< 1.7 mmol/L, *p* for interaction = 0.023). The odds of albuminuria were significantly higher in subjects with HR than in those without HR (grade 1, odds ratio [OR] = 1.57, 95% CI: 1.08, 2.29, *p* = 0.019; grade 2, OR = 2.02, 95% CI: 1.28, 3.18, *p* = 0.002; grade 3, OR = 2.12, 95% CI: 0.99, 4.55, *p* = 0.053). In the subgroup analyses, the association between HR grades 1 and 2 and albuminuria was stronger in subjects with higher triglycerides levels (≥ 1.7 mmol/L), but for grade 3 HR, this correlation was stronger in subjects with lower triglyceride levels (< 1.7 mmol/L, *p* for interaction = 0.014).

**Conclusion:**

HR was positively correlated with albuminuria in hypertensive Chinese adults. This correlation was more remarkable when the population was stratified by triglycerides levels and smoking status. HR can be used as an indicator of early renal injury.

**Supplementary Information:**

The online version contains supplementary material available at 10.1186/s12886-023-02807-6.

## Introduction

Approximately one-third of people in China suffer from hypertension [1]. Chronic kidney disease (CKD), which is both a cause and complication of hypertension, affects approximately 119.5 million people in China and has become a serious public health concern [2].

Dipstick proteinuria tests are a common and inexpensive method for screening renal diseases, but they are not sufficiently precise [3]. A total of 10.5% of dipstick proteinuria‑negative subjects still have renal injury [4]. Albuminuria is a biomarker of early kidney injury. It is not only a risk factor for end-stage renal disease, progressive CKD and acute kidney injury [5] but also an independent predictive factor for cardiovascular and all-cause mortality [3]. According to an epidemiological study, more people in rural China than urban China have albuminuria [2]. However, laboratory tests for albuminuria are not widely used in rural China and some developing countries due to their high cost.

Retinal blood vessels are similar in anatomy and physiology to vessels in other end organs. Retinal microvascular abnormalities (RMAs), especially vasoconstriction, stenosis and enhanced arterial reflex, are considered the main pathological features of hypertension and are closely related to the left ventricular failure, stroke, nephropathy, and cardiovascular disease observed in hypertensive patients [6]. Retinal blood vessels can be observed directly through a fundus examination, which represents a noninvasive and convenient method for assessing end-organ damage.

The relationship between RMA and albuminuria has been reported in previous studies, but their results are controversial. For example, Bao et al. found that a lower retinal arteriovenous ratio was associated with higher albuminuria [7], but no positive association was found between these parameters in Masaidi’s study [8]. Previous studies were mainly carried out in Europe and America. However, in the Chinese population, especially in rural areas, the correlation between RMA and albuminuria has not been fully clarified, and this group is very different from European and American people in terms of diet structure, lifestyle and economic conditions. In addition, previous studies have not evaluated in detail the possible effect modifiers.

The purpose of this study was to assess the correlation between albuminuria and hypertensive retinopathy (HR) in hypertensive adults in rural China. These data may provide a theoretical basis for the use of fundus examination to screen for early renal injury in hypertensive subjects.

## Material and methods

### Study design and population

This is a cross-sectional study. Data used in this analysis were obtained from the China Stroke Primary Prevention Trial, a multicenter, double-blind and randomized clinical study performed in rural China. The purpose of the China Stroke Primary Prevention Trial was to compare the efficacy of folic acid combined with enalapril and enalapril alone in preventing stroke in Chinese hypertensive adults [9]. The study was carried out in 32 communities in Anhui and Jiangsu Provinces beginning in May 2008, and subjects were followed up for 5 years. We conducted a cross-sectional analysis of data obtained from the last follow-up in 2013. A total of 3,860 adults with primary hypertension aged between 45 and 75 years old who had laboratory tests for albuminuria were included in the study. Among these individuals, 3,121 underwent fundus photography. The procedures of the study were in accordance with the Helsinki Declaration. The Ethics Committee of the Institute of Biomedicine at Anhui Medical University approved all procedures used in the study. All participants provided informed consent before the study began. The study was registered at http://clinicaltrials.gov/, NCT number: NCT00794885.

### Data collection

#### Demographic data

The general information related to the participants was collected through a questionnaire. Past history of illness, current medication use, smoking and drinking habits were recorded in detail. Smoking and drinking habits were each categorized into 3 levels: current smoking or drinking, former smoking or drinking and never smoking or drinking. Smoking at least 1 cigarette per day for 6 months was defined as current smoking. Drinking at least once a week for 6 months was defined as current drinking. The blood pressure of all participants was measured in the sitting position after at least 10 min of rest. The measurements were made 3 times at two-minute intervals, and the results were averaged. Hypertension was defined as systolic blood pressure (SBP) ≥ 140 mmHg, diastolic blood pressure (DBP) ≥ 90 mmHg, or the use of antihypertensive drugs [9], and diabetes mellitus was defined as having a history of diabetes, using antidiabetic medications, or fasting blood glucose (FBG) ≥ 7.0 mmol/L in laboratory tests. Body height and body weight were measured, and body mass index (BMI) was calculated by dividing weight (kg) by height^2^ (m^2^).

#### Laboratory tests

All participants provided spot urine samples, and fasting venous blood was drawn. The samples were tested in the laboratory of the National Clinical Research Center for Kidney Disease, Nanfang Hospital, Guangzhou, China. The test methods were as follows. Triglyceride (TG), total cholesterol (TCHO), serum creatine (Scr), FBG, homocysteine (HCY) and uric acid (UA) levels were tested using automatic clinical analyzers (Beckman Coulter); Hyperuricemia was defined as UA ≥ 360 μmol/L in females and 420 μmol/L in males [10]; the estimated glomerular filtration rate (eGFR) was calculated according to the Chronic Kidney Disease Epidemiology Collaboration creatinine equation [11]; serum folate levels were measured using chemiluminescent immunoassays (New Industrial); methylenetetrahydrofolate reductase (MTHFR) C677T gene polymorphisms (CC, CT and TT) were detected using an ABI Prism 7900HT sequence detection system (Life Technologies); urinary albumin levels were measured using an automatic protein analyzer (BN II; Dade Behring); urinary creatinine levels were tested using an automatic biochemical analyzer (Dimension RxL Max; Dade Behring); the urinary albumin to creatinine ratio (UACR) was calculated as the urinary albumin level divided by the urinary creatinine level (mg/g), and a UACR ≥ 30 mg/g was defined as albuminuria [2].

#### Evaluation of retinopathy

Macula-centered and nonmydriatic fundus pictures were taken by fundus cameras (Canon CR-2 AF, Japan, Kowa nonmyd 7, Japan and Topcon TRC-NW8, Japan) and randomly assessed by 4 eye doctors in a double-masked manner. The assessment of the effect of hypertension on retinal vessels was based on the Keith-Wagener-Barker stages of HR [12, 13] (Supplemental Table 1). Consistency checks were conducted, and the results were found to be reliable (kappa values ranged from 0.71 to 0.95) [14].

### Statistical analysis

**Fig. 1:**
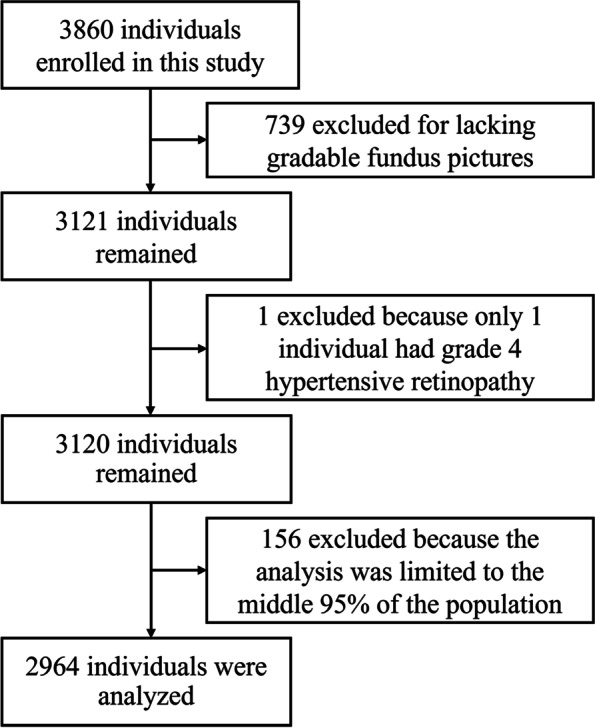
Flow chart of the participants

All statistical analyses were conducted using EmpowerStats (X&Y Solutions, Inc. Boston, MA) software and R (version 3.4.3). Among 3121 participants, only one female was diagnosed with grade 4 HR. Due to an insufficient sample size, subjects with grade 4 were not grouped for analysis. Then, according to the continuous distribution data of albuminuria, we selected the middle 95% of the population, and a total of 2964 subjects were included in the study (Fig. 1). The mean ± the standard deviation and median (interquartile range) are used to express continuous variables, while frequencies are used to express categorical variables. Differences in population characteristics by HR grades were compared using ANOVA tests, Kruskal–Wallis test, or chi-square tests, accordingly. The correlation between albuminuria (as a continuous variable or a categorical variable) and HR was evaluated using multiple linear regression analyses, with adjustment for age, sex, and BMI in model I and age, sex, BMI, SBP, DBP, MTHFR C677T polymorphisms, TCHO, TG, FBG, eGFR, folate, HCY, smoking status, alcohol consumption, and the use of antihypertensive medicine in model II. Stratified analyses were performed to evaluate the relationship between albuminuria (as a continuous variable or a categorical variable) and HR in the following different subgroups: age (< 65 or ≥ 65 years old), sex, BMI (< 24 or ≥ 24 and < 28 or ≥ 28 kg/m^2^), treatment group (enalapril or enalapril + folic acid), SBP (< 140 or ≥ 140 mmHg), TCHO (< 5.2 or ≥ 5.2 mmol/L), TG (< 1.7 or ≥ 1.7 mmol/L), FBG (< 7.0 or ≥ 7.0 mmol/L), HCY (< 10 or ≥ 10 and < 15 or ≥ 15 μmol/L), hyperuricemia (yes or no), and alcohol consumption and smoking status (never, former, or current). Tests of interactions were performed to assess whether a variable influenced the effect of HR on UACR and albuminuria. The outcomes were expressed as the β or odds ratio (OR) with 95% confidence intervals (CIs). HR was transformed into continuous variables for the trend test. *P* < 0.05 was considered statistically significant.

## Results

HR was found in 76.6% (*n* = 2, 271) of the 2964 participants, including 58.9% (*n* = 1, 747) who were categorized as grade 1, 15.0% (*n* = 446) who were categorized as grade 2, and 2.6% (*n* = 78) who were categorized as grade 3.

The baseline characteristics of the subjects are shown in Table 1. There were 1070 males and 1894 females, and the mean age of the participants was 63.5 ± 7.3 years old. The median (interquartile range) UACR level was 10.5 (7.2, 17.1) mg/g, and albuminuria was found in 11.1% (*n* = 330). Compared with the normal subjects, the subjects with HR had a higher BMI (*p* = 0.035), SBP (*p* = 0.006), DBP (*p* = 0.002), UA (*p* = 0.005), Scr (*p* =  < 0.001) and UACR (*p* = 0.003), a lower eGFR (*p* = 0.007), and a higher proportion of individuals with diabetes (*p* = 0.045) and albuminuria (*p* = 0.004).

**Table 1:** Characteristics of the subjects by hypertensive retinopathy group^a^

Characteristics	Overall	Normal	Grade 1	Grade 2	Grade 3	*p*
**N**	2964	693	1747	446	78	
**Age, years**	63.5 ± 7.3	63.4 ± 7.1	63.7 ± 7.5	62.4 ± 6.7	63.5 ± 7.4	0.008
**Male, N (%)**	1070 (36.1)	228 (32.9)	649 (37.2)	164 (36.8)	29 (37.2)	0.254
**BMI, kg/m**^**2**^	25.7 ± 3.8	25.6 ± 3.7	25.6 ± 3.8	26.1 ± 3.9	26.0 ± 4.4	0.035
**Treatment Group, N (%)**						0.590
Enalapril	1255 (50.7)	293 (51.1)	744 (51.0)	190 (50.3)	28 (42.4)	
Enalapril + folic acid	1222 (49.3)	280 (48.9)	716 (49.0)	188 (49.7)	38 (57.6)	
**MTHER C677T, N (%)**						0.668
CC	673 (22.9)	164 (23.8)	381 (22.1)	108 (24.3)	20 (25.6)	
CT	1469 (50.0)	344 (50.0)	872 (50.6)	211 (47.4)	42 (53.8)	
TT	794 (27.0)	180 (26.2)	472 (27.4)	126 (28.3)	16 (20.5)	
**Smoking status, N (%)**						0.725
Never	2094 (71.1)	494 (71.9)	1236 (71.1)	307 (69.1)	57 (74.0)	
Former	284 (9.6)	65 (9.5)	170 (9.8)	40 (9.0)	9 (11.7)	
Current	569 (19.3)	128 (18.6)	333 (19.1)	97 (21.8)	11 (14.3)	
**Alcohol consumption, N (%)**						0.659
Never	2089 (73.8)	509 (76.3)	1207 (72.7)	317 (73.7)	56 (74.7)	
Former	168 (5.9)	33 (4.9)	106 (6.4)	26 (6.0)	3 (4.0)	
Current	575 (20.3)	125 (18.7)	347 (20.9)	87 (20.2)	16 (21.3)	
**Diabetes, N (%)**	68 (2.3)	11 (1.6)	43 (2.5)	9 (2.0)	5 (6.6)	0.045
**Medication use, N (%)**						
Antihypertensive treatment	2797 (95.1)	643 (93.9)	1657 (95.5)	426 (95.9)	71 (92.2)	0.179
**SBP, mmHg**	137.7 ± 17.5	135.9 ± 16.6	137.9 ± 17.6	139.1 ± 18.2	140.4 ± 18.2	0.006
**DBP, mmHg**	83.0 ± 10.7	82.2 ± 10.1	82.8 ± 10.7	84.4 ± 11.6	84.9 ± 11.5	0.002
**Laboratory results**						
TCHO, mmol/L	5.4(4.7,6.0)	5.3(4.7,6.0)	5.4(4.8,6.0)	5.5(4.7,6.1)	5.4(4.7,6.1)	0.810
TG, mmol/L	1.5(1.1,2.2)	1.4(1.1,2.2)	1.5(1.1,2,2)	1.6(1.1,2,2)	1.5(1.0,2.4)	0.252
FBG, mmol/L	5.8(5.4,6.5)	5.8(5.4,6.4)	5.8(5.4,6.5)	5.8(5.3,6.6)	5.9(5.4,8.9)	0.148
UA, μmol/L	314.0(55.8,74.3)	303(259,367)	319(269,379)	315(267,380)	319.5(266,384.5)	0.005
Scr, μmol/L	64.3(55.8,74.3)	62.0(54.5,71.9)	64.8(56.4,75.1)	65.2(56.0,75.1)	64.6(52.8,71.1)	< 0.001
Folate, ng/mL	14.8(10.8,19.8)	14.8(11.0,19.6)	14.7(10.6,19.9)	14.8(10.8,19.3)	15.6(11.4,20.6)	0.709
HCY, μmol/L	12.0(10.1,14.8)	12.0(10.0,14.2)	12.2(10.2,14.9)	12.0(10.2,15.1)	12.0(10.6,13.8)	0.046
eGFR, mL/min/1.73 m^2^	92.6(83.6,99.1)	93.7(86.3,99.7)	92.0(83.0,98.7)	92.3(83.2,99.4)	92.7(82.6,99.1)	0.007
UACR, mg/g	10.5(7.2,17.1)	10.1(7.1,16.8)	10.4(7.2,16.5)	10.9(7.7,18.5)	13.7(7.8,22.6)	0.003
**Albuminuria, N (%)**	330 (11.1)	55 (7.9)	200 (11.4)	62 (13.9)	13 (16.7)	0.004

*BMI* Body mass index, *MTHER* Methylenetetrahydrofolate reductase, *SBP* Systolic blood pressure, *DBP* Diastolic blood pressure, *TCHO* Total cholesterol, *TG* Triglyceride, *FBG* Fasting blood glucose, *UA* Uric acid, *Scr* Serum creatinine, *HCY* Homocysteine, *eGFR* Estimated glomerular filtration rate, *UACR* Urinary albumin to creatinine ratio

^a^For continuous variables, values are presented as the mean ± standard deviation or median (interquartile range). For categorical variables, values are presented as frequencies

The results of multivariable regression analyses between HR and UACR are shown in Table 2. In the unadjusted model, HR was associated with higher UACR levels than were found in those with a normal fundus test (grade 1, β = 1.53, 95% CI: 0.06, 3.01, *p* = 0.042; grade 2, β = 2.69, 95% CI: 0.69, 4.68, *p* = 0.008; grade 3, β = 5.61, 95% CI: 1.69, 9.54, *p* = 0.005). After adjusting for age, sex and BMI, this trend was sustained (grade 1, β = 1.72, 95% CI: 0.26, 3.17, *p* = 0.021; grade 2, β = 3.15, 95% CI: 1.18, 5.12, *p* = 0.002; grade 3, β = 5.88, 95% CI: 2.01, 9.76, *p* = 0.003). After adjusting for age, sex, MTHFR C677T genotypes, BMI, SBP, DBP, FBG, TG, TCHO, eGFR, folate, HCY, smoking status, alcohol consumption, and the use of antihypertensive medicine, the trend was also sustained (grade 1, β = 1.42, 95% CI: -0.12, 2.95, *p* = 0.070; grade 2, β = 2.62, 95% CI: 0.56, 4.67, *p* = 0.013; grade 3, β = 5.17, 95% CI: 1.13, 9.20, *p* = 0.012).

**Table 2:** The association between hypertensive retinopathy and UACR^a^

Hypertensive retinopathy	N	UACR, mg/g Median (interquartile range)	Unadjusted model		Model I		Model II	
			**β (95% CI)**	***p***	**β (95% CI)**	***p***	**β (95% CI)**	***p***
Normal	693	10.1 (7.1,16.8)	ref		ref		ref	
Grade 1	1747	10.4 (7.2,16.5)	1.53 (0.06,3.01)	0.042	1.72 (0.26,3.17)	0.021	1.42 (-0.12,2.95)	0.070
Grade 2	446	10.9 (7.7,18.5)	2.69 (0.69,4.68)	0.008	3.15 (1.18,5.12)	0.002	2.62 (0.56,4.67)	0.013
Grade 3	78	13.7 (7.8,22.6)	5.61 (1.69,9.54)	0.005	5.88 (2.01,9.76)	0.003	5.17 (1.13,9.20)	0.012
*p* for trend				< 0.001		< 0.001		0.001

*UACR* Urinary albumin to creatinine ratio, *CI* Confidence interval

^a^Model I was adjusted for age, sex, and body mass index; Model II was adjusted for age, sex, body mass index, systolic blood pressure, diastolic blood pressure, methylenetetrahydrofolate reductase C677T polymorphisms, total cholesterol, triglycerides, fasting blood glucose, estimated glomerular filtration rate, folate, homocysteine, smoking status, alcohol consumption, and the use of antihypertensive drugs

The results of multivariable regression analyses between HR and albuminuria are shown in Table 3. In the unadjusted model, HR was associated with higher odds of albuminuria than was found in subjects with a normal fundus test (grade 1, OR = 1.50, 95% CI: 1.10, 2.05, *p* = 0.011; grade 2, OR = 1.87, 95% CI: 1.28, 2.75, *p* = 0.001; grade 3, OR = 2.32, 95% CI: 1.20, 4.47, *p* = 0.012). This trend was sustained after adjusting for variables in model I (grade 1, OR = 1.51, 95% CI: 1.10, 2.07, *p* = 0.011; grade 2, OR = 1.99, 95% CI: 1.35, 2.95, *p* < 0.001; grade 3, OR = 2.37, 95% CI: 1.22, 4.61, *p* = 0.011) and after adjusting for variables in model II (grade 1, OR = 1.57, 95% CI: 1.08, 2.29, *p* = 0.019; grade 2, OR = 2.02, 95% CI: 1.28, 3.18, *p* = 0.002; grade 3, OR = 2.12, 95% CI: 0.99, 4.55, *p* = 0.053).

**Table 3:** The association between hypertensive retinopathy and albuminuria^a^

Hypertensive retinopathy	N	Albuminuria N (%)	Unadjusted model		Model I		Model II	
			**OR (95% CI)**	***p***	**OR (95% CI)**	***p***	**OR (95% CI)**	***p***
Normal	693	55 (7.9)	ref		ref		ref	
Grade 1	1747	200 (11.4)	1.50 (1.10,2.05)	0.011	1.51 (1.10,2.07)	0.011	1.57 (1.08,2.29)	0.019
Grade 2	446	62 (13.9)	1.87 (1.28,2.75)	0.001	1.99 (1.35,2.95)	< 0.001	2.02 (1.28,3.18)	0.002
Grade 3	78	13 (16.7)	2.32 (1.20,4.47)	0.012	2.37 (1.22,4.61)	0.011	2.12 (0.99,4.55)	0.053
*p* for trend				< 0.001		< 0.001		0.002

*OR* Odds ratio, *CI* Confidence intervals

^a^Model I was adjusted for age, sex, and body mass index; Model II was adjusted for age, sex, body mass index, systolic blood pressure, diastolic blood pressure, methylenetetrahydrofolate reductase C677T polymorphisms, total cholesterol, triglycerides, fasting blood glucose, estimated glomerular filtration rate, folate, homocysteine, smoking status, alcohol consumption, and the use of antihypertensive drugs

The results of stratified analyses between HR and the UACR are shown in Table 4. There were significant interactions when data were stratified by TG (*p* for interaction = 0.023) and smoking status (*p* for interaction = 0.014). The correlation between UACR and HR was stronger in current smokers (grade 1, β = 2.5, 95% CI: -1.22, 6.23; grade 2, β = 4.64, 95% CI: -0.13, 9.42; grade 3, β = 18.73, 95% CI: 8.47, 29.00). There were strong correlations between UACR and HR grades 1 and 2 in subjects with TGs ≥ 1.7 mmol/L (grade 1, β = 3.17, 95% CI: 0.53, 5.81; grade 2, β = 4.7, 95% CI: 1.25, 8.15). However, in regard to grade 3, a strong correlation between UACR and HR was found in subjects with TG < 1.7 mmol/L (β = 7.59, 95% CI: 2.8, 12.37).

**Table 4:** The association between hypertensive retinopathy and the UACR by subgroup^a^

**Subgroup**	**N**	**UACR, Median (interquartile range)**	**Hypertensive retinopathy groups**				***p* for interaction**
			**Normal**	**Grade 1**	**Grade 2**	**Grade 3**	
				**β (95% CI)**	**β (95% CI)**	**β (95% CI)**	
**Sex**							0.498
Male	1070	8.3 (6.1,13.2)	ref	2.54(-0.16,5.25)	4.3(0.77,7.82)	8.17(1.15,15.19)	
Female	1893	11.8 (8.3,18.7)	ref	0.97(-0.9,2.83)	1.97(-0.57,4.52)	3.48(-1.45,8.41)	
**Age, years**							0.107
< 65	1769	10 (7.1,15.7)	ref	0.95(-0.97,2.86)	2.78(0.26,5.29)	1.56(-3.58,6.7)	
≥ 65	1178	11.4 (7.5,19.3)	ref	2.27(-0.28,4.82)	2.24(-1.33,5.81)	10.5(3.94,17.06)	
**BMI, kg/m2**							0.220
< 24	1042	10.3 (7.1,16.6)	ref	-0.62(-3.17,1.93)	2.19(-1.31,5.69)	4.3(-2.53,11.12)	
≥ 24, < 28	1178	10.3 (7.2,16.7)	ref	1.97(-0.42,4.35)	3.5(0.29,6.72)	6.15(-0.44,12.75)	
≥ 28	728	11.2 (7.6,18.3)	ref	3.15(-0.11,6.41)	1.48(-2.72,5.67)	6.54(-1.4,14.49)	
**Treatment group**							0.380
Enalapril	1255	10.3 (7.1,16.5)	ref	0.37(-1.69,2.43)	1.85(-0.91,4.61)	7.55(1.53,13.58)	
Enalapril + folic acid	1222	10 (7,15.7)	ref	2.72(0.31,5.13)	4(0.76,7.25)	5.06(-0.81,10.94)	
**SBP, mmHg**							0.402
< 140	1686	9.5 (6.8,14.9)	ref	1.2(-0.67,3.07)	2.97(0.39,5.56)	1.74(-3.76,7.24)	
≥ 140	1246	11.9 (8.1,20.2)	ref	2.05(-0.55,4.64)	2.47(-0.88,5.83)	8.49(2.51,14.48)	
**TG, mmol/L**							0.023
< 1.7	1722	10.1 (7,15.9)	ref	0.21(-1.64,2.06)	1.19(-1.34,3.73)	7.59(2.8,12.37)	
≥ 1.7	1201	11.1 (7.7,18.6)	ref	3.17(0.53,5.81)	4.7(1.25,8.15)	0.13(-7.04,7.31)	
**TCHO, mmol/L**							0.353
< 5.2	1259	10.2 (7.2,16.5)	ref	2.06(-0.1,4.22)	2.7(-0.34,5.74)	8.94(3.18,14.7)	
≥ 5.2	1642	10.7 (7.2,17.5)	ref	0.92(-1.24,3.07)	2.64(-0.16,5.45)	1.76(-3.86,7.38)	
**FBG, mmol/L**							0.251
< 7.0	2393	10 (7.1,15.7)	ref	0.89(-0.68,2.46)	2.42(0.28,4.55)	3.87(-0.76,8.51)	
≥ 7.0	508	13.8 (8.4,23.4)	ref	4.22(-0.76,9.2)	3.17(-3.13,9.46)	9.2(0.04,18.37)	
**HCY, μmol/L**							0.882
< 10	686	10.5 (7.7,17.9)	ref	0.65(-2.4,3.7)	0.8(-3.37,4.96)	9.38(0.31,18.44)	
≥ 10, < 15	1554	10.4 (7.2,16.8)	ref	1.55(-0.54,3.64)	2.44(-0.43,5.31)	3.67(-1.52,8.86)	
≥ 15	678	10.3 (7.1,16.8)	ref	1.75(-1.67,5.17)	3.77(-0.57,8.12)	5.4(-4.26,15.06)	
**Hyperuricemia**							0.432
No	2303	10.5 (7.2,17.2)	ref	1.72(0.04,3.41)	2.13(-0.17,4.43)	5.61(1.03,10.2)	
Yes	624	10.3 (7.1,16.7)	ref	0.82(-2.87,4.5)	4.05(-0.6,8.71)	3(-5.59,11.58)	
**Smoking status**							0.014
Never	2094	11.3 (7.8,18.3)	ref	0.67(-1.09,2.43)	1.77(-0.62,4.16)	2.46(-2.12,7.03)	
Former	284	8.9 (6.5,14.1)	ref	3.54(-2.08,9.17)	4.79(-2.65,12.24)	-5.3(-20.25,9.65)	
Current	569	8.7 (6.2,14.1)	ref	2.5(-1.22,6.23)	4.64(-0.13,9.42)	18.73(8.47,29)	
**Alcohol consumption**							0.447
Never	2089	11.1 (7.8,18.1)	ref	1.05(-0.68,2.77)	2.14(-0.2,4.47)	4.11(-0.38,8.6)	
Former	168	9 (6.7,19.5)	ref	4.56(-4.69,13.81)	3.29(-8.53,15.11)	11.04(-17.93,40.01)	
Current	575	9 (6.2,14.5)	ref	1.43(-2.05,4.9)	4.24(-0.29,8.77)	5.93(-3.35,15.2)	

*UACR* Urinary albumin to creatinine ratio, *CI* Confidence intervals, *BMI* Body mass index, *SBP* Systolic blood pressure, *TG* Triglyceride, *TCHO* Total cholesterol, *FBG* Fasting blood glucose, *HCY* Homocysteine

^a^Adjusted for age, sex, body mass index, systolic blood pressure, diastolic blood pressure, methylenetetrahydrofolate reductase C677T polymorphisms, total cholesterol, triglycerides, fasting blood glucose, estimated glomerular filtration rate, folate, homocysteine, smoking status, alcohol consumption, and the use of antihypertensive drugs

The results of stratified analyses between HR and albuminuria are shown in Table 5. There was a significant interaction when data were stratified by TG (*p* for interaction = 0.014). There was a strong correlation between albuminuria and HR grades 1 and 2 in subjects with TGs ≥ 1.7 mmol/L (grade 1, OR = 2.58, 95% CI: 1.36, 4.9; grade 2, OR = 3.62, 95% CI: 1.75, 7.48). However, in regard to grade 3, a strong correlation between albuminuria and HR was found in subjects with TG < 1.7 mmol/L (OR = 2.7, 95% CI: 1.09, 6.73).

**Table 5:** The association between hypertensive retinopathy and albuminuria by subgroup^a^

**Subgroup**	**N**	**Albuminuria,** **N (%)**		**Hypertensive retinopathy groups**			***p* for interaction**
			**Normal**	**Grade 1**	**Grade 2**	**Grade 3**	
				**OR (95% CI)**	**OR (95% CI)**	**OR (95% CI)**	
**Sex**							0.780
Male	1070	94 (8.8)	ref	1.91(0.89,4.08)	2.34(0.96,5.71)	3.33(0.78,14.24)	
Female	1893	235 (12.4)	ref	1.49(0.96,2.32)	2.05(1.2,3.5)	1.78(0.71,4.49)	
**Age, years**							0.147
< 65	1769	163 (9.2)	ref	1.53(0.9,2.6)	2.33(1.27,4.29)	0.89(0.23,3.45)	
≥ 65	1178	164 (13.9)	ref	1.69(0.98,2.91)	1.59(0.78,3.24)	4.1(1.52,11.06)	
**BMI, kg/m**^**2**^							0.391
< 24	1042	105 (10.1)	ref	1.1(0.58,2.07)	2.26(1.07,4.8)	1.29(0.29,5.63)	
≥ 24, < 28	1178	134 (11.4)	ref	1.77(0.95,3.3)	2.17(1.03,4.6)	2.75(0.79,9.59)	
≥ 28	728	90 (12.4)	ref	2.13(0.99,4.57)	1.49(0.58,3.85)	3.36(0.85,13.27)	
**Treatment group**							0.184
Enalapril	1255	123 (9.8)	ref	1.06(0.64,1.76)	1.45(0.77,2.7)	2.48(0.85,7.24)	
Enalapril + folic acid	1222	121 (9.9)	ref	2.48(1.27,4.82)	3.07(1.42,6.68)	2.8(0.85,9.23)	
**SBP, mmHg**							0.785
< 140	1686	137 (8.1)	ref	1.56(0.9,2.69)	2.37(1.24,4.55)	1.66(0.45,6.06)	
≥ 140	1246	189 (15.2)	ref	1.63(0.96,2.77)	1.81(0.95,3.45)	2.54(0.94,6.83)	
**TG, mmol/L**							0.014
< 1.7	1722	158 (9.2)	ref	1.06(0.65,1.72)	1.28(0.68,2.39)	2.7(1.09,6.73)	
≥ 1.7	1201	166 (13.8)	ref	2.58(1.36,4.9)	3.62(1.75,7.48)	1.08(0.22,5.38)	
**TCHO, mmol/L**							0.237
< 5.2	1259	126 (10.0)	ref	1.77(0.96,3.28)	2.59(1.24,5.41)	3.86(1.31,11.38)	
≥ 5.2	1642	195 (11.9)	ref	1.43(0.88,2.32)	1.7(0.94,3.06)	1(0.31,3.22)	
**FBG, mmol/L**							0.693
< 7.0	2393	223 (9.3)	ref	1.47(0.95,2.27)	2.09(1.24,3.52)	1.98(0.71,5.54)	
≥ 7.0	508	98 (19.3)	ref	1.94(0.88,4.28)	1.78(0.68,4.64)	2.97(0.89,9.95)	
**HCY, μmol/L**							0.947
< 10	686	75 (10.9)	ref	1.1(0.51,2.34)	1.63(0.65,4.13)	2.57(0.53,12.44)	
≥ 10, < 15	1554	165 (10.6)	ref	1.61(0.97,2.68)	1.99(1.06,3.73)	1.76(0.62,5.01)	
≥ 15	678	82 (12.1)	ref	2.24(0.89,5.66)	2.54(0.88,7.37)	4(0.63,25.19)	
**Hyperuricemia**							0.528
No	2303	251 (10.9)	ref	1.66(1.09,2.51)	1.85(1.1,3.1)	2.45(1.04,5.78)	
Yes	624	72 (11.5)	ref	1.65(0.64,4.24)	3.16(1.11,8.99)	1.41(0.23,8.66)	
**Smoking status**							0.217
Never	2094	245 (11.7)	ref	1.39(0.91,2.13)	1.77(1.05,2.99)	1.58(0.63,3.95)	
Former	284	29 (10.2)	ref	5.37(0.63,45.8)	4.81(0.45,51.81)	0(0,Inf)	
Current	569	55 (9.7)	ref	1.84(0.68,4.96)	3.17(1.04,9.67)	5.7(1,32.68)	
**Alcohol consumption**							0.861
Never	2089	242 (11.6)	ref	1.58(1.02,2.45)	2.01(1.18,3.41)	1.94(0.8,4.68)	
Former	168	27 (16.1)	ref	3.84(0.67,22.08)	4.06(0.45,36.69)	4.07(0.04,411.17)	
Current	575	52 (9)	ref	1.15(0.46,2.84)	2.12(0.74,6.09)	2.84(0.48,16.98)	

*OR* Odds ratio, *CI* Confidence intervals, *BMI* Body mass index, *SBP* Systolic blood pressure, *TG* Triglyceride, *TCHO* Total cholesterol, *FBG* Fasting blood glucose, *HCY* Homocysteine

^a^Adjusted for age, sex, body mass index, systolic blood pressure, diastolic blood pressure, methylenetetrahydrofolate reductase C677T polymorphisms, total cholesterol, triglycerides, fasting blood glucose, estimated glomerular filtration rate, folate, homocysteine, smoking status, alcohol consumption, and the use of antihypertensive drugs

## Discussion

The prevalence of albuminuria and CKD was 9.4% and 10.8%, respectively in the general Chinese population [2]. Since hypertension is a risk factor for albuminuria and CKD [2], this figure could be higher among hypertension patients. Our results show that among subjects with hypertension, the prevalence of albuminuria was 11.1%, which is slightly higher than that in the general population. Such a high proportion makes CKD a serious public health problem in China, and early detection is becoming particularly important.

Retinopathy and nephropathy are both microvascular diseases and target organ damage observed in hypertension, and there is a definite correlation between them. CKD has been associated with various ocular diseases, such as cataracts, glaucoma, diabetic retinopathy, age-related macular degeneration and vision loss due to various causes [15]. In Bao’s study, a lower arteriovenous ratio was associated with a higher risk of albuminuria and CKD [7]. Huang et al. found that the central retinal artery equivalent was negatively correlated with the UACR in a Chinese population with hypertension [16]. Shantha et al. indicated that HR of any grade had moderate accuracy in predicting microalbuminuria [17, 18]. Vadala et al. concluded that albuminuria was negatively correlated with the index of superficial foveal and parafoveal vessel density in hypertensive patients [19].

In this study, there was a significant correlation between HR grade and albuminuria in hypertensive subjects. In subjects with grade 1 HR, compared with the normal subjects, the UACR increased by 1.42 mg/g, and the odds of albuminuria increased by 57%; in subjects with grade 2 HR, the UACR increased by 2.62 mg/g, and the odds of albuminuria increased by 102%; and in subjects with grade 3 HR, the UACR increased by 5.17 mg/g, and the odds of albuminuria increased by 112%. This suggests that with the aggravation of HR, albuminuria is more obvious, and renal damage is more serious. As a systemic disease, the effects of hypertension on the retinal microvascular and renal microvascular are often parallel [19]. When the glomerular capillary endothelium is damaged, albumin leaks from the blood into the urine. As the structural characteristics of glomerular capillaries are similar to those of systemic vessels, the appearance of albuminuria reflects not only kidney injury but also systemic vascular endothelium abnormalities and atherosclerosis [20, 21]. RMAs are also closely related to endothelial dysfunction, inflammation and arteriosclerosis [14, 22]. This can explain the internal relationship between retinopathy and kidney injury. Therefore, HR can be used as a screening tool for albuminuria and early renal injury.

However, contradictory findings have also been reported. For example, in Bao’s study, the central retinal artery equivalent, central retinal vein equivalent and arteriovenous ratio were not associated with albuminuria and reduced renal function in hypertensive patients [7], and Masaidi et al. reported that there were no significant correlations between the arteriovenous ratio and either eGFR or microalbuminuria in hypertensive patients [8]. These differences may be due to the use of different study populations, the inclusion criteria or adjustment variables applied or the methods used to evaluate the fundus.

In the stratified analyses, a stronger correlation between the UACR and HR was found in current smokers and subjects with higher TG levels, except for in those with grade 3 HR, and the correlations between UACR and grade 3 HR were stronger in subjects with lower TG levels.

Smoking can induce inflammation, oxidative stress and renal fibrosis, leading to kidney injury [23]. Similarly, damage can also occur in the retina. For example, smoking is associated with thinning of the retinal nerve fiber layer [24], choroidal neovascularization and retinal pigment epithelium damage [25]. Recent studies have shown that smoking can reduce vascular density and expand the foveal avascular zone of the retina, indicating that smoking can lead to RMA [26]. Therefore, it is well understood that the correlation between HR and albuminuria is stronger in current smokers. The TG level is a risk factor for endothelial dysfunction [27], arteriosclerosis and cardiovascular disease [28], and TG-lowering therapy is an effective method to reduce cardiovascular and cerebrovascular events [28]. High TG levels are positively associated with nephropathy [29] and retinopathy [30, 31]. This finding can explain the results that the correlation between HR grades 1 and 2 and albuminuria is stronger in subjects with higher TG levels. Interestingly, however, in regard to grade 3, the correlation between HR and albuminuria was stronger in subjects with TG < 1.7 mmol/L. Some studies have also shown that TG is protective against retinopathy [32]. However, due to the small sample size of patients with grade 3 HR in the current study, the reliability of the results may also be affected, and the specific mechanism needs to be further studied.

The strength of this study is that we found a positive correlation between HR and albuminuria in hypertensive adults in rural China. We evaluated the possible effect modifiers in detail and found that this correlation was stronger when the population was stratified by TG levels and smoking status. This finding provides evidence for exploring the relationship between smoking, TG and RMA in the future. It also provides a theoretical basis for focusing on screening and follow-up in people with a possibility of kidney damage, such as smokers with HR. Retinal blood vessels, the only blood vessels that can be directly observed in vivo, are an important marker of systemic vascular disease. If ophthalmologists better understand the relationship between fundus diseases and systemic diseases, they will be able to detect many potential systemic diseases earlier. In the CSPPT cohort, we have been continuously following up the patients for many years to collect more endpoint data for exploration and analysis. We hope to obtain more meaningful information about the correlation between HR and other microvascular diseases by studying this cohort.

There are several limitations to this study. First, because of the limitations of cross-sectional studies, causality could not be obtained. Second, since the fundus pictures were obtained by a nonmydriatic fundus camera, peripheral lesions may have been missed. HR often occurs around the optic disc and macula, and nonmydriatic fundus examination can obtain most of the information. Third, we evaluated RMA based on the Keith-Wagener-Barker stage instead of quantitative parameters. This evaluation of clinical signs may lack objectivity, especially in evaluating “generalized retinal arteriolar narrowing” in grade 1 HR and “definite focal narrowing” in grade 2 HR. To improve the consistency, we trained the evaluators in detail before the study and conducted a consistency test [14]. In addition, although measurements of the arteriovenous ratio can be used to quantify arteriolar narrowing [7, 8, 16], this method lacks indicators, such as focal narrowing, arteriovenous nipping, hemorrhage and exudate, that can better reflect the severity of retinal atherosclerosis and destruction of the blood–retina barrier. Fourth, the sample size of patients with grade 3 HR was small, which may affect the reliability of the results of this group. Because most of the patients in this study were treated with antihypertensive therapy, malignant hypertension was rare. Fifth, our study did not exclude other preexisting kidney diseases, which may affect the accuracy of the UACR results. This should be taken into account when interpreting the results of the study. To reduce the possible impact of renal function on the outcome, we included eGFR in the adjusted variables for multiple regression analysis.

In conclusion, we found that HR was positively correlated with albuminuria in hypertensive adults. This correlation was stronger in current smokers. The correlation between HR grades 1 and 2 and albuminuria was stronger in subjects with higher TG levels, but in regard to grade 3 HR, this correlation was stronger in subjects with lower TG levels. RMA associated with hypertension can be used as an indicator of early renal injury. These results highlight the need for albuminuria screening in hypertensive patients complicated with HR to ensure early detection of CKD.

## Supplementary Information

**Additional file 1: Supplementary Table 1.** The Keith-Wagener-Barker classification for hypertensive retinopathy.

## Data Availability

The data that support the findings of this study are not publicly available because sharing these data might compromise the privacy of the research participants, but the data are available from the corresponding author upon reasonable request.

## Abbreviations

BMI: Body mass index
CI: Confidence intervals
CKD: Chronic kidney disease
DBP: Diastolic blood pressure
eGFR: Estimated glomerular filtration rate
FBG: Fasting blood glucose
HCY: Homocysteine
HR: Hypertensive retinopathy
MTHFR: Methylenetetrahydrofolate reductase
OR: Odds ratio
RMA: Retinal microvascular abnormalities
SBP: Systolic blood pressure
Scr: Serum creatine
TG: Triglyceride
TCHO: Total cholesterol
UA: Uric acid
UACR: Urinary albumin to creatinine ratio
