# Supplementary material for: Positive correlation between hypertensive retinopathy and albuminuria in hypertensive adults
Source: BMC Ophthalmol. 2023 Feb 13;23:66. doi: 10.1186/s12886-023-02807-6 (PMC9923927; doi:10.1186/s12886-023-02807-6)
Supplement: Supplementary file 1 — Additional file 1: Supplementary Table 1. The Keith-Wagener-Barker classification for hypertensive retinopathy. [file 12886_2023_2807_MOESM1_ESM.docx]

**Supplementary table 1 The Keith-Wagener-Barker classification for hypertensive retinopathy**

| **Grade** | **Features** |
| --- | --- |
| **Normal** | No detectable positive signs |
| **1** | Mild generalized retinal arteriolar narrowing |
| **2** | Definite focal narrowing and arteriovenous nipping |
| **3** | Signs of grade 2 retinopathy plus retinal hemorrhages, exudates and cotton wool spots |
| **4** | Severe grade 3 retinopathy plus papilledema |
